# 3D Scaffolds Fabrication via Bicomponent Microgels Assembly: Process Optimization and *In Vitro* Characterization

**DOI:** 10.3390/mi13101726

**Published:** 2022-10-12

**Authors:** Iriczalli Cruz-Maya, Vincenzo Guarino

**Affiliations:** Institute of Polymers, Composites and Biomaterials, National Research Council of Italy, Mostra d’Oltremare, Pad. 20, V. le J. F. Kennedy 54, 80125 Naples, Italy

**Keywords:** scaffolds, alginate, gelatin, hMSC, biocompatibility

## Abstract

In the last decade, different technological approaches have been proposed for the fabrication of 3D models suitable to evaluate *in vitro* cell response. Among them, electro fluid dynamic atomization (EFDA) belonging to the family of electro-assisted technologies allows for the dropping of polysaccharides and/or proteins solutions to produce micro-scaled hydrogels or microgels with the peculiar features of hydrogel-like materials (i.e., biocompatibility, wettability, swelling). In this work, a method to fabricate 3D scaffolds by the assembly of bicomponent microgels made of sodium alginate and gelatin was proposed. As first step, optical and scanning electron microscopy with the support of image analysis enabled to explore the basic properties of single blocks in terms of correlation between particle morphology and process parameters (i.e., voltage, flow rate, electrode gap, and needle diameter). Chemical analysis via ninhydrin essays and FTIR analysis confirmed the presence of gelatin, mostly retained by physical interactions into the alginate network mediated by electrostatic forces. *In vitro* tests confirmed the effect of biochemical signals exerted by the protein on the biological response of hMSCs cultured onto the microgels surface. Hence, it is concluded that alginate/gelatin microgels assemblies can efficiently work as 3D scaffolds able to support *in vitro* cells functions, thus providing a friendly microenvironment to investigate *in vitro* cell interactions.

## 1. Introduction

In the current scenario of tissue engineering, hydrogels are widely used to guide local interactions between cells and materials, as a function of specific cues dictated by morphology and biochemical composition [1]. Indeed, their three-dimensional network coupled with hydrophilicity/swelling properties make them ideal materials to support adhesion, spreading, and cell growth *in vitro* [2]. Moreover, the possibility to influence water uptake as a function of the cell culture conditions (i.e., salt concentration) allows facilitating the exchange of nutrients across the polymeric network, providing an *in vitro* microenvironment that mimics that of the native extracellular matrix (ECM), supporting cell proliferation and differentiation [1]. In the last five years, a relevant interest was gained by the use of microgels—i.e., hydrogels with extended exchange surface area due to their sizes at the microscale—with enhanced swelling properties and permeability, able to improve cell interaction *in vitro* and related events (i.e., adhesion, proliferation, migration, and differentiation) [2]. They are generally fabricated by using biopolymers from a natural source to minimize problems of scarce biocompatibility/toxicity during cell culture.

Among them, natural polysaccharides such as alginates are preferred due to their good biocompatibility, as well as the ease to be processed in different forms, i.e., injectable systems, foams and/or porous scaffolds, or fibrous membranes [3]. From a chemical point of view, alginates can be classified as block copolymers by the combination of β-D-mannuronate (M-block) and α-L-guluronate (G-block) units. Ionic crosslinking through G-blocks and divalent cations (i.e., Ca^2+^, Cu^2+^, Mg^2+^, Sr^2+^) allows forming a stable 3D network suitable to host *in vitro* cells for different biological approaches [4,5,6].

Despite the recognized biocompatibility of alginates, it has been noticed that alginate by itself does not provide a biochemical signal able to stimulate cell adhesion. Due to the increasing interest in the use of alginate-based materials, alginates have been recently modified by simple routes that involve the chemical or physical binding of peptides or other bioactive compounds (i.e., growth factors, morphogenic proteins) able to improve cell interface [7,8]. In this way, electrically charged proteins such as collagen, keratin, gelatin, or silk may offer a great opportunity to form stable complexes with anionic polyelectrolites such as alginates [9]. Among them, gelatin—a natural protein derived from animal origin due to the denaturation of collagen—is universally recognized due to its biocompatibility, biodegradable, and non-toxicity in its lack of immune response in the body [10]. The innate presence of RGD (arginine-glycine-aspartic) sequences—that are commonly involved in integrin-mediated cell adhesion—along their macromolecules guarantees unmatched outcomes in terms of adhesion and cell proliferation [11,12].

Herein, electro fluid dynamic atomization (EFDA)—a versatile and low-cost process for the fabrication of highly customizable devices [13,14] in terms of size, morphology, and composition—has been successfully used to fabricate microgels—made of alginate and gelatin—with improved bio recognition for *in vitro* studies [15,16,17]. In particular, EFDA was complemented with a simple post-processing method that allowed to assembly microgels until to form 3D porous scaffolds. An accurate optimization of the process conditions was performed in order to modulate morphological and biochemical properties. Then, biological response of hMSC was assessed to validate their use as 3D scaffolds for tissue engineering.

## 2. Materials and Methods

### 2.1. Materials

Low viscosity (LV, 250 cps) sodium alginates (SA) from brown algae, gelatin type B (225 g bloom), 1-ethyl-3-(3-dimethylaminopropyl)carbodiimide hydrochloride (EDC), N-hydroxysulfosuccinimide (N-NHS), and anhydrous calcium chloride (CaCl_2_) were purchased by Sigma Aldrich, Milan, Italy. Deionized water was used for the preparation of the solution. In addition, 18- and 27-gauge (G) needles for the atomization process were purchased by BD, USA. As for biological assays, human mesenchymal stem cells (hMSCs, SCC034), Eagle’s alpha minimum essential medium (α-MEM), fetal bovine serum (FBS), antibiotic solution (streptomycin 100 µg/mL and penicillin 100 U/mL), L-glutamine, and cell proliferation Kit II (XTT, Roche) were purchased by Sigma Aldrich, Milan, Italy.

### 2.2. Fabrication of Microgels

**Figure 1:**
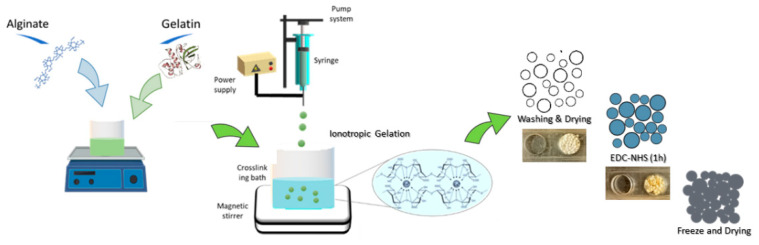
Schematic representation of the preparation 3D scaffolds via EFDA/freeze drying.

Sodium alginate (SA) aqueous solution was prepared at 2% *w*/*v* for the fabrication of sodium alginate microgels (Alg). In the case of bicomponent microgels (Alg/Gel), gelatin type A (1% *w*/*v*) was added to the SA solution and stirred 30 min at 50 °C to completely solubilize the gelatin. The microgels were fabricated by using a commercial machine (NF500 MECC, Fukuoka, Japan) equipped with a tailor-made magnetic stirrer working as collector in agreement with previous studies [18]. The EFDA parameters were optimized to control the size and shape of microgels. Briefly, Alg and Alg/Gel solutions were placed in a 5 mL syringe connected to a power supply—applied voltage 25 kV—by a metallic needle (18 Ga). The solution was pumped by imposing a feed rate equal to 1.0 mL/h. Tip-to-collector distance was set at 150 mm. Droplets were collected into a CaCl_2_ solution at a concentration of 1.1% under magnetic stirring to trigger the ionotropic interaction between SA and Gelatin in aqueous solution (Figure 1). After a rest time (1 h) in 2-(*N*-morpholino)ethanesulfonic acid (MES) buffer containing a mixture of EDC-NHS (molar ratio equal 2) [19] into circular molds—i.e., 120 mm as diameters, 2 mm as thickness—samples were frozen at −18 °C for 24 h and finally dried overnight.

### 2.3. Morphological Characterization

Morphology of Alg and Alg/Gel was evaluated by optical microscopy (DM750, Leica, Germany). Optical images were used to qualitatively and quantitatively estimate microgel sizes via image analysis (Image J, 1.47; NIH, Bethesda, MD, USA). Results were reported as mean ± standard deviation (SD). The surface morphology of microgels was investigated by scanning electron microscopy (SEM, Quanta FEG 200 FEI, Eindhoven, The Netherlands) working at low voltage electron emission (2 kV) into a low vacuum range (e.g., chamber pressure < 10^−2^ Pa) to avoid any sample pre-treatment via conductive metal deposition.

### 2.4. Chemical Characterization

Chemical composition of microgels was examined by means of Fourier transform infrared spectroscopy coupled with attenuated total reflectance technique (ATR-FTIR). The spectra were acquired in the spectral region between 4000 and 400 cm^−1^. The analysis was performed using the Origin software (OriginPro 8 SR0; OriginLab Corporation, Northampton, MA, USA).

To qualitatively detect the content of amino groups by gelatin content in the microgels, ninhydrin assay was also employed. A ninhydrin solution was prepared by dissolving 0.2 g of ninhydrin in 100 mL of ethanol. About 5 mg of microgels were placed in a tube with ninhydrin solution and heated at 80 °C for 15 min. The reaction of ninhydrin with amino groups induces a staining change from white to a deep purple color, as a function of the amount of amino groups.

### 2.5. In Vitro Biocompatibility

*In vitro* assays were conducted by using human mesenchymal stem cells (hMSCs). hMSCs were cultured in a 75 cm^2^ cell culture flask in Eagle’s alpha minimum essential medium (α-MEM) supplemented with 10% FBS, antibiotic solution (streptomycin 100 µg/mL and penicillin 100 U/mL) and 2 mM of L-glutamine, incubated at 37 °C in a humidified atmosphere with 5% CO_2_ and 95% air. hMSCs from 4–6 passage were used for cell proliferation assays.

Three-dimensional assemblies placed into multi-wells were washed and sterilized in ethanol (70%) for 30 min, washed three times with PBS, and finally incubated with cell culture media that was retired after 30 min. hMSCs were seeded at 1 × 10^4^ to perform cell adhesion and proliferation assays with Cell Proliferation Kit II (XTT, Roche, Basel, Switzerland). For cell adhesion, after 24 h, medium was taken. To remove the unattached cells, samples were washed. Then, 100 µL of fresh medium with 50 µL of XTT was added to each sample to incubate for 4 h in standard conditions. After incubation time, the supernatant was collected and placed into a microplate reader to measure the absorbance at 450 nm. Results are presented as percentage of cell adhesion respective to the cell culture plate. For cell proliferation, XTT assay kit was performed after 1, 3, and 7. Briefly, at each time point, cell culture medium was removed and changed by 100 µL of fresh medium with XTT working solution as indicated above. After 4 h the supernatant was collected, and absorbance measured at 450 nm using microplate reader.

## 3. Results and Discussion

In the last years, SA-based hydrogels have been widely studied as 3D platforms for different biomedical applications. In particular, alginate beads have been proposed as a good alternative to study cell behavior, providing a suitable platform for anchorage-dependent cells [20]. However, alginate-based materials have shown some biological drawbacks, due to the lack of adhesive properties that limit the cell recognition [21,22]. To overcome this limitation, the addition of bioactive proteins such as gelatin can currently be considered a promising route to improve cell adhesion in order to more efficiently reproduce biological microenvironment of natural tissues [23]. Hence, it was optimized the fabrication of them by a discontinuous process based on the dripping of charged droplets under the application of electric forces. In this case, droplet shape and structure were stabilized by a gelation mechanism triggered by ionotropic interaction in the collecting bath [17,24]. An accurate setting of material parameters including solution properties (e.g., polymer concentration, molecular weight, viscosity) and process conditions (i.e., voltage, flow rate, distance between electrodes, needle diameter) enabled to fabricate microgels with tailored morphological features (i.e., size, shape), and easy to be functionalized by biochemical signals to support cell interactions [20]. Herein, microgels were also assembled to form hydrogel-like scaffolds with improved features for biological use: interconnected pores generated by adjacent microgels can guide cell ingrowth and tissue formation, while large surface/volume ratio and short diffusion distance can more efficiently support mass transport and nutrients exchange, with a key role on long-term survival of cells [25,26].

### 3.1. Microgels Characterization

**Figure 2:**
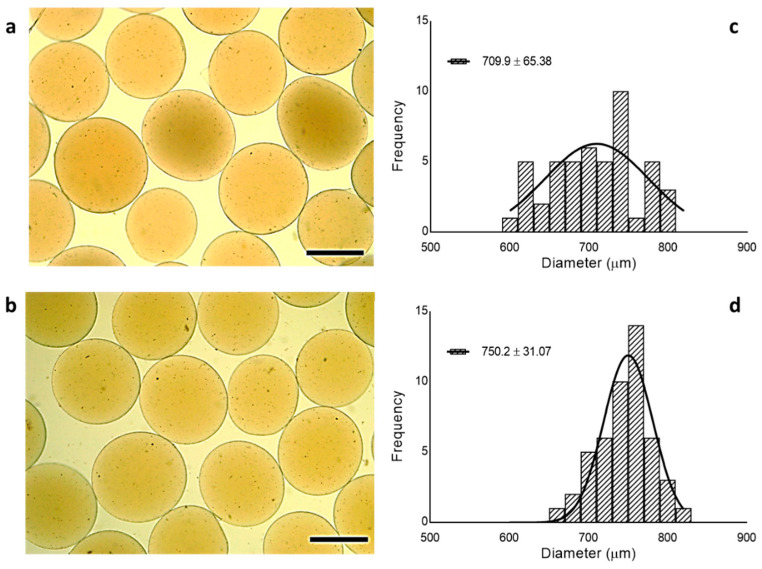
Optical images of (**a**) Alg and (**b**) Alg/Gel microgels (Scale bar: 500 µm) and relative diameter distribution (**c**,**d**).

Alg and Alg/Gel morphology was firstly analyzed via optical microscopy (Figure 2a,b). Optical images show a spherical shape in both cases, and average diameters from 709.9 ± 65.38 µm to 750.2 ± 31.07 µm with some differences in terms of diameter distribution (Figure 2c,d). Indeed, Alg microgels were formed by an ionotropic gelation mechanism triggered by the interaction with divalent ions such as Ca^2+^ into the bath. Once droplets have reached the bath, ion diffusion occurs by a promotion of physical bonds among the guluronic fraction of the polymer chains, with the formation of not homogeneous regions until the formation of microgels. While alginate tends to instantaneously form gel-like phases onto the surface, a gradient variously crosslinked zones is formed due to the different ion extent, related to the diffusion time from the core towards the gelled zone [27]. In the case of Alg/Gel microgels, polar groups of protein tend to influence the ion diffusion mechanism through the alginate network, thus contributing to reducing structural heterogeneity, also with effects on the diameter distribution.

**Figure 3:**
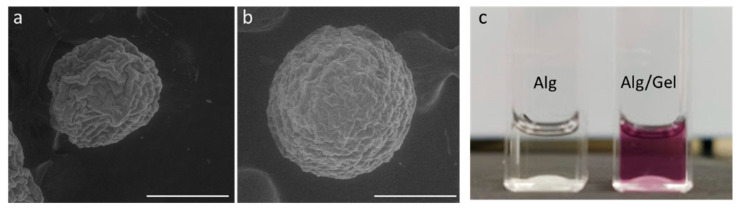
SEM images of (**a**) Alg and (**b**) Alg/Gel microgels (scale bar: 300 µm). (**c**) Ninhydrin staining test to detect the presence of gelatin into the bicomponent microgels.

Further investigations of the surface morphology were assessed by SEM analysis performed in low vacuum mode to minimize the shrinkage effects ascribable to high pressure decay (Figure 3). SEM images highlighted a rough surface independently of the presence of gelatin in the microgels. After vacuum stabilization, a remarkable shrinkage of the microgel volume was recognized in the case of Alg (≈50%) (Figure 3a). This effect is mitigated by the presence of gelatin that reduces this effect by about 30% (Figure 3b).

**Figure 4:**
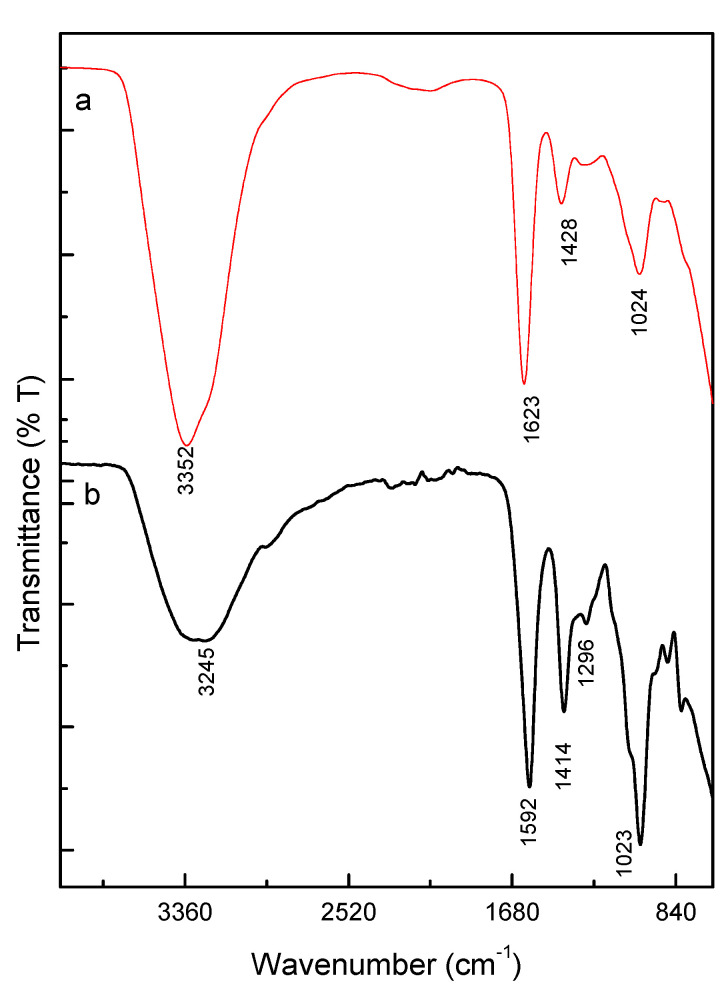
ATR-FTIR spectrum of (**a**) Alg and (**b**) Alg/Gel microgels.

To confirm the presence of the gelatin into the microgels, ATR-FTIR spectroscopic analyses were performed. Figure 4 shows the spectra of Alg and Alg/Gel microgels, where it is possible to recognize the characteristic peaks of sodium alginate including 3352 cm^−1^, 1623 cm^−1^, and 1428 cm^−1^ attributed to the asymmetric stretching vibration and symmetric stretching vibration of -COO group, respectively [28]. The peak at 1024 cm^−1^ corresponds to the stretching vibrations of C-O bond [29,30]. The amide groups characteristic of proteins as gelatin are recognized in the regions of 1650 cm^−1^ and 1540 cm^−1^ attributed to Amide I (C=O and C-N stretching vibration) and Amide II [31]. For Alg/Gel microgels, the amide group bands were slightly shifted to 1542 cm^−1^ and 1414 cm^−1^ [32]. The bands corresponding to amide III, N-H bending vibration, and C-N stretching vibration are located at 1296 cm^−1^, confirming the presence of gelatin into microgels. Notably, the -OH stretching band is present in both spectra around 3352 cm^−1^. In the case of Alg/Gel microgels, a wider peak was recorded due to the higher hydrogen bonding, that is slightly shifted, due to the stretching vibration of O-H bonded to N-H, in agreement with experimental evidences previously reported for similar systems [33,34].

### 3.2. In Vitro Characterization of 3D Scaffolds

In this view, the integration of a bio-instructive proteins such as gelatin, strictly embedded into the alginate network, may corroborate the biocompatible functions of scaffolds, improving the cell material interface over the intrinsic lack of alginate biorecognition [21].

**Figure 5:**
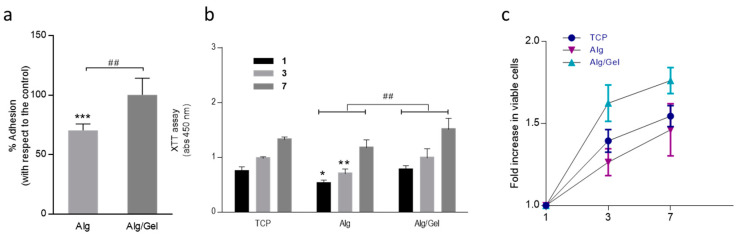
*In vitro* tests on Alg and Alg/Gel assembled scaffolds: (**a**) Adhesion and (**b**) proliferation (statistical difference * *p* < 0.05, ** *p* < 0.01, *** *p* < 0.001 respect to the control (TCP); ^##^ *p* < 0.01 statistical difference between Alg and Alg/Gel); (**c**) cell viability fold increase in time.

Accordingly, the biological response of hMSC on Alg/Gel and Alg scaffolds was investigated. In Figure 4, all the data of biocompatibility tests were summarized. Figure 4a shows the cell adhesion percentage respect to the cell culture plate after 24 h in *in vitro* culture. Alg/Gel presented near 100% of cell adhesion comparable to the cell culture plate, differently to the alginate microgels, which showed around 70% of cell adhesion (Figure 5a). This is not surprising because gelatin—derived from collagen by hydrolysis—is a non-antigenic protein including bioactive sequences as RGD, naturally present in the ECM, that enhance the integrin-mediated cell adhesion. This is in agreement with a large variety of experimental studies reported in literature, that combine gelatin with either natural or synthetic polymers [35,36,37,38,39,40]. Moreover, the incorporation of gelatin into hydrogel-like materials such as alginates allows to preserve, to a large extent, the peculiar transport properties in terms of water uptake of the matrix. In combination with the presence of macropores formed by the pseudo-sintering of microgels, this certainly concurs to create a pattern of morphological/biochemical/biophysical cues that promote relevant benefits in terms of cell viability.

Cell proliferation was evaluated via XTT assay (Figure 5b). Results showed a significant improvement of cell response in the presence of Alg/Gel respect to Alg microgels, directly ascribable to the contribution of gelatin on hMSC. Indeed, Alg/Gel ones enable the creation of a bio-instructive microenvironment able to better sustain cell proliferation in time respect to Alg, due to the integration and the good packing of bioactive protein. Moreover, gelatin has been widely used in tissue engineering and drug delivery; however, the high solubility of gelatin makes necessary the use of crosslinking agents (i.e., glutaraldehyde, genipin) that may present some cytotoxic effect [19,41]. Contrariwise, the proposed approach allows to entrap gelatin into the alginate network by the use of a non-toxic physical process—i.e., ionotropic gelation corroborated by the effect of electrostatic forces—that assures an efficient the retention of the protein into the alginate network, minimizing the cytotoxic effect and promoting the cell proliferation over a week in *in vitro* culture (Figure 5b). Once assembled to form 3D scaffolds, it originates a platform able to mimic the chemical and 3D structure of ECM. This is confirmed by several fold increase of cells number detected after 7 days onto Alg/Gel assemblies, in comparison with 2D cell culture plate (Figure 5c). This can be directly ascribable to the high surface area of single units (i.e., microgels) composing the scaffold, that concur to prevent stationary growth phase, opening new possibilities to study *in vitro* cell processes for longer times [42,43].

Notably, similar technological approaches have been recently validated for the fabrication of different types of microgels with tunable properties for *in vitro* applications. For instance, over the improvements of surface and mechanical properties, gelatin methacryloyl hydrogel microgels were functionalized via selective bioactive molecules to improve cell interaction and differentiation mechanisms via imaging applications [44]. Li et al. designed a tissue-like 3D structure by assembling PEG/gelatin microgels via covalent bonding (4-arm PEG-NHS as crosslinker) [45] to support the *in vitro* functionalities of human bone marrow mesenchymal stem cells (hBMSCs), upregulating chondrogenic markers in both gene (Sox-9, Aggrecan, COMP, Col2a1, Col1a1) and glycosaminoglycan (GAG) expression levels. Basiri et al. optimized the fabrication of microgels assemblies composed of gelatin–phenol conjugate and silk fibroin working as angiogenesis promotive system suitable to monitor via imaging/tracking VEGF delivery in ischemic muscles [46].

Accordingly, with these previous studies, Alg/Gel scaffolds can offer an optimal compromise to design bioinspired analogues of 3D cell microenvironment: alginate provides a stable and biocompatible network with improved transport properties while gelatin working as the bioactive component promotes adhesion and cell proliferation, assuring good stability condition. Lastly, interconnected microporous network of microgel assembly contributes to accelerating fluid transport and nutrient transfer, supporting cell viability, also facilitating cell infiltration and, in perspective, vascularization, so showing a broad pattern of potential applications from the scaffold for tissue repair/regeneration to cell/molecular carrier for signal and gene upregulation.

## 4. Conclusions

In this work, a top-down approach to fabricate Alg/Gel microgels was integrated to a simple methodology to produce 3D scaffolds by their random assembly. First, the proposed experimental setup is simple and does not require complex steps. The use of a high voltage electric field as driving force for the polymer solution dripping is not invasive and does not alter the chemical functionalities of both biopolymers used. Rather then, the use of an ion bath as collector allows to trigger ionotropic gelation mechanisms that are fundamental to efficiently entrap gelatin into the microgels, not altering the innate properties of the polymer network, suitable for an efficient transport of fluids and molecules in the cell culture. Herein, it is demonstrated that gelatin uniformly dispersed into the 3D assembly concurs to promote cell adhesion and proliferation, relative to that without protein. In this view, Alg/Gel microgels assemblies allow reproducing the typical conditions required to supply cell functions in a simulated biological microenvironment of natural tissues, suitable as instructive scaffolds for tissue engineering or as a highly customizable 3D model to predict in vivo-like response of cells for preclinical studies.

## Data Availability

All datasets generated for this study are included in the article.
